# Fresh Fish: Observation up Close in Late Seventeenth-Century England

**DOI:** 10.1098/rsnr.2019.0051

**Published:** 2021-09-20

**Authors:** Didi van Trijp

**Affiliations:** Leiden University Centre for the Arts in Society, Leiden University, Arsenaalstraat 1, 2311 CT, Leiden, The Netherlands

**Keywords:** Royal Society, *Historia piscium*, natural history, practice, experience, observation

## Abstract

The traditional view of London's Royal Society as a closed circle has been subject to revision in the past decades. Historians have shown the considerable extent to which the Fellows of the Society drew on a broad range of men of practice for their respective skill sets. This article offers an in-depth analysis of the contributions of fishermen and fishmongers to the creation of natural knowledge. It centres on the *Historia piscium* (Oxford, 1686), written by Francis Willughby and John Ray, and its surrounding sources. This natural history of fishes aspired to give a concise and precise overview of species, and to uncover the divine order in which they were created. While men of practice contributed to this project in multiple ways, their first-hand observations carried particular weight. Through their cumulative experience of working with fish they saw a great number of living species, rather than the dried exemplars that naturalists would usually consult in cabinets of curiosities, or the indirect evidence that images might present. This article examines what kind of exchanges took place between fishermen and fishmongers on the one hand and Fellows on the other, and where, how and why these were incorporated into the fish book. In so doing, it also aims to qualify the value attached to direct (natural historical) observation in the socio-cultural context of late seventeenth-century England.

## Introduction

Fish were part and parcel of daily life in early modern England. An annotated copy of Francis Willughby and John Ray's *Historia piscium* (Oxford, 1686) in the archives of the Royal Society accentuates this, as some of its marginalia specify where in London one might have chanced upon which species of fish.^1^ They reveal that lampreys could be seen shining in the water of the Thames before fishermen hauled them up in wicker nets, while London shops displayed a selection of dabs.^2^ A dolphin—at that time still considered a fish—taken ‘in our Channell; very smooth like polisht marble a long snout with 2 rows of teeth on each side, very little Eyes & c. about 4 feet long’ could be encountered ‘at the Ship Tavern at Butcher Row's end near Temple Bar’.^3^ The swim bladder of the cod counted as a ‘very luxurious’ dish in the city.^4^ Furthermore, any strange fishes caught in the Thames were brought to the Lord Mayor's home.^5^ Despite their ubiquitous presence, however, fish were also somewhat elusive: these ‘slippery denizens’ of the water were difficult to capture, and once caught they promptly began to falter and spoil.^6^ Where and how, then, could one establish solid knowledge about these rather unstable objects of inquiry?

**Figure 1.**
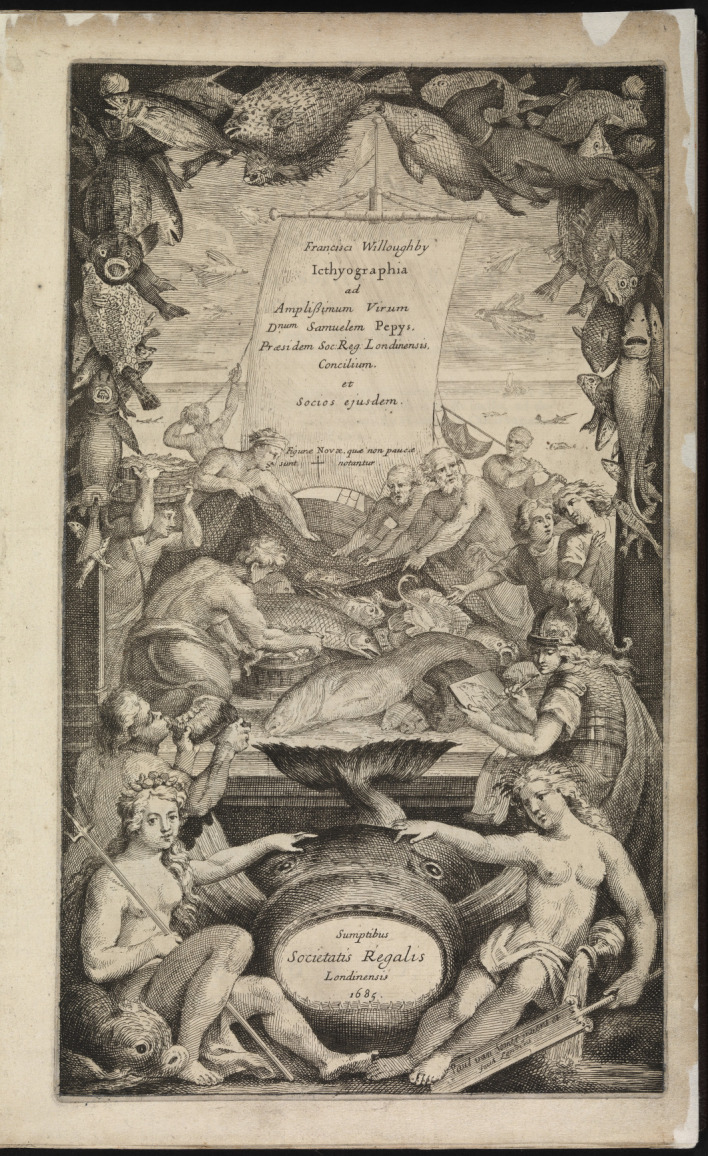
Paul van Somer II, title page of *Icthyographia* (1685). (© The Royal Society.)

Questions such as these occupied Fellows of the Royal Society during the preparation, production and publication of the *Historia piscium*. This book, based on the research of Willughby (1635–1672) and Ray (1627–1705), strove to offer accurate accounts of all fish hitherto known, and to do so in an orderly manner.^7^ The resulting work was a voluminous and rather expensive work in folio format encompassing hundreds of species descriptions and almost 200 sumptuous full-page copperplate engravings bound together in the appendix.^8^ The engraved title page (figure 1) made by the Dutch painter and printmaker Paul van Somer II (1644–1698) begs a close look.^9^ Set against the backdrop of an Arcadian fishing port, several people tend to the arrival of fresh fish, announced by a herald blowing a large conch shell.^10^ Fishermen in loincloths haul in their nets. Two men dressed in tunics examine the scene, one of whom gestures at the catch. Just below them, a female figure in a helmet, possibly a reference to Minerva, the Roman goddess of wisdom and the arts, draws the specimen that is set before her. A garland of fish lines the sides and top of the frontispiece; the pufferfish, turbot and hound shark are copied from the engraved plates of the book.^11^ These depictions are decidedly different from the dolphin, taken from classical iconography, that adorns the lower left corner of the engraving. The colossal fish in the foreground, containing the book's imprint in its gaping mouth, is rendered in a similarly stylized manner. To the right of this creature, a female figure reposes on a jug from which water is pouring, adding to the sense of flow and movement of the scene. All in all, the title page evokes a sense of exuberance and abundance. Considering that frontispieces of early modern works of natural history and philosophy often present a visual programme of a book's contents,^12^ this one brings together various sources for knowledge about fish: classical accounts, illustration and first-hand observation.

The materials drawn upon for the *Historia piscium* were rich indeed and encompassed earlier natural historical works, travel accounts, objects in cabinets of curiosities, drawings bound together in books, loose drawings and observations shared in letters, as well as observations made by the authors during their own field work.^13^ This article investigates one of the sources displayed on the title page: namely, those practically engaged with fish such as fishermen and fishmongers, whose contributions have yet to be thoroughly researched.^14^ The nature and extent of the contributions of these practical men can be inferred both from the *Historia piscium* itself, and from other source materials related to the book and its authors, such as natural historical manuscripts, minutes of Royal Society meetings and letters to and from the Fellows.^15^

The article consists of three parts. The first part explains how *Historia piscium* took shape as a collaborative project of the Royal Society in the socio-cultural context of knowledge production particular to late seventeenth-century England. The second part takes us to fishing ports and fish markets and discusses how fishermen and fishmongers provided fresh fish for natural historical study, and why this was so important. The third part addresses how these practical men contributed to the identification of, and distinction between, species and sometimes remarked on specific behaviour. The article concludes that the emphasis placed on direct observation as a requisite for establishing an accurate account of species left considerable space for the experience of people of practice in the production of natural knowledge.

## A wider cast

The variety of sources displayed on the title page of the *Historia piscium* is also reflected in the text itself, such as in the discussion of the peculiar way in which the salmon every so often leaps out of the water:

The salmon constantly presses forward against the stream, and when it encounters in its ascent an enclosure or another obstacle of this kind, it seizes, after it has bent its body in a circle, its tail with its mouth, and, while it holds fast to this [i.e. its tail], it, releasing [its grip] again, with great force, leaps across it. Author of *De natura rerum* with *Gesner*. We have heard multiple times of many fishermen that this happens continually. That salmon are most agile in jumping we confirm willingly, and our daily experience confirms this: but what is told about the seizing of the tail seems to us less plausible.^16^

Several layers of observation come together in this passage. It begins with an ancient account, possibly taken from a poem by Lucretius, as cited in Conrad Gessner (1516–1565).^17^ While this is illustrative of the extent to which Willughby and Ray drew on the works of Renaissance authors,^18^ we also see that they did not take such accounts at face value. Willughby and Ray verified this account not once but in multiple instances, and not with one but with many fishermen—who, furthermore, confirmed that they saw this happening all the time. This in itself, however, still did not settle the matter of the salmon's strange behaviour. While Willughby and Ray's own, daily experiences confirmed the tenor of the report—namely that salmon are nimble jumpers—they remained sceptical about its specifics, particularly the manner in which the salmon gripped and released its tail, which they had not seen themselves. The *Historia piscium* contains many passages like this, which collate observations from both past and present sources before concluding with the authors' own verdict on the matter.

Because their history of fishes was a joint project, it can be difficult to attribute certain statements or ideas to either Willughby or Ray with certainty. Such attempts are further complicated by Willughby's early death, 14 years before the project came to completion, which left Ray to turn their notes into a book. Historians have long debated which of them contributed most to the work;^19^ while some claim that it was Willughby, who was of higher social standing and employed Ray, others take Ray's seniority in age as a mark of his authority.^20^ Aside from these differences, they had much in common, including being educated at the University of Cambridge. Willughby and Ray were also both elected to the Royal Society, in 1661 and 1667 respectively; the engraved title page of their fish book displays this affiliation. As Sachiko Kusukawa has shown, the Fellows of the Society helped to amass relevant material for the book and evaluated whether certain observations merited inclusion. Tancred Robinson and Martin Lister took active roles in bringing the *Historia piscium* to publication.^21^ They, as well as other Fellows and friends, passed their own observations to Willughby and Ray in letters written in English, parts of which were then translated into Latin and included.^22^ One result of this multitude of contributors is that it is often difficult to know exactly who contributed what to the *Historia piscium* and when. It is nonetheless clear that the Society played an important role in the production of the book and that, as Kusukawa has stated, ‘the Fellows’ collective engagement with it fundamentally shaped the way it was published'.^23^

We can also recognize the book as a product of the Royal Society in its insistence on knowledge derived from direct experience with the object of study.^24^ In the epilogue to the *Historia piscium*, Ray contended that it would ‘bring across exactly these things which were either observed by ourselves and our friends, or which had proper witnesses and authors, worthy of our trust’.^25^ While earlier authors counted as credible past witnesses, their written observations were, ideally, corroborated with those of contemporary ones. Indications of direct observation are present in the fish book in various ways. Willughby and Ray, for example, added ‘I have seen’ (*vidi*) or ‘we have seen’ (*vidimus*) to certain species descriptions. In other cases, they punctuated statements with appeals to ‘experience’ (*experientia*), as in the case of the salmon. The exact meaning of this term was far from fixed in the early modern period.^26^ While Peter Dear argued that, in the early years of the Royal Society, ‘experience’ was used for witnessing or participating in a particular, singular event tied to a specific moment, rather than for generalized statements on universal phenomena (in the Aristotelian sense of the term), the term is used in both senses within the *Historia piscium*.^27^

It is well known that emphasis on first-hand observation (for which the terms *observatio* and *autopsia* gained currency) rose steadily from the early sixteenth century onwards.^28^ While the Royal Society's adherence to the philosophical programme of Francis Bacon (1561–1626) may well have been somewhat overstated, the Society's foregrounding of direct experience as the foundation of natural knowledge does owe much to Bacon's work.^29^ Bacon had stated that experience of nature might be gained through hunting, husbandry, gardening, shepherding, animal breeding and travelling, among other things.^30^ ‘The materials for the intellect’, he wrote, ‘are so widely spread out that they ought to be sought out and gathered in (as if by agents and merchants) from all sides.’^31^ He also held that one would be ‘forever tossed and turned on the waves of experience’ when pursuing it without clear course.^32^ What was needed, then, were philosophers with literate, learned experience, examining nature step by step in an orderly manner.^33^ Deborah E. Harkness has contended, however, that Bacon's precepts for obtaining true and certain natural knowledge were not altogether different from the daily vernacular science practised in the streets of Elizabethan London.^34^

Fishermen and fishmongers, as attentive observers of nature, were consulted broadly throughout the early modern period. Renaissance naturalists such as Guillaume Rondelet and Pierre Belon, for example, conversed with fishermen on their observations of Mediterranean marine life in addition to perusing learned books, a practice that Florike Egmond has referred to as ‘fieldwork once removed’.^35^ Gessner, too, stated that he benefited from the knowledge of fishermen, and attributed a higher value to first-hand observation than he did to natural knowledge of the textual kind.^36^ Monica Azzolini has shown how, in Rome, naturalists like Johannes Faber (1574–1629) made ample use of a plurality of oral sources consisting of, among others, fishermen, merchants and servants, when investigating beached whales.^37^

These interactions take on a new meaning, in an English context at least, with the surge of scientific societies in the seventeenth century. Membership of such a group, which was usually restricted to those of the upper classes, considerably heightened one's credibility.^38^ When discussing Faber's report on the whale in the *Historia piscium*, for example, Ray noted that the Roman was a member of the Accademia dei Lincei.^39^ In the Royal Society, the existing convention of assigning reliability to those of higher social status remained in place when observing and interpreting natural phenomena.^40^ This did not mean, however, that status was the sole criterion of credibility.^41^ While those from a genteel background were generally seen as trustworthy, they were also considered prone to bending their observations to fit with preconceived ideas.^42^ Philippa Hellawell has argued that credibility was not the exclusive prerogative of one particular social group, but that it could be shared, albeit attributed in various degrees, among people of various backgrounds.^43^ Felicity Henderson has submitted that the Royal Society, as an institution, relied on ‘the activities and expertise of wider penumbra of individuals’ than those of the Fellows themselves.^44^ Certain individuals within the Society itself blurred social boundaries, such as Robert Hooke (1635–1703). Despite being employed as Curator of Experiments—regarded as a lesser position because of the paid labour involved—he was also elected Fellow and took part in natural philosophical debates.^45^

Experiments held a special place in the early Royal Society. Bacon had contended that they served to deliberately seek out a certain experience, as opposed to experience derived from ‘accident’—allotting an active role to the observer, rather than a passive one.^46^ While the Fellows seem to have had their own approaches to the meaning and use of experiments, it is clear that several of them took to performing these to understand nature's intriguing properties.^47^ About fish, they wondered: did they breathe? How did these creatures move in the water? How did they spawn, and how long could they go without food? Meeting minutes in the Journal Books of the early 1660s reveal that the Society's Operator, tasked with facilitating experiments and making inquiries, was ordered several times to collect and keep fish for experiments.^48^ He was also instructed to ask fishermen how long they could keep their fish alive without feeding them.^49^ Furthermore, the minutes indicate that ‘all those [present], that had the opportun[it]y, were desired to make several Experiments in several fish, concerning their growth’.^50^

Although the precise set-up of these experiments is not always disclosed in the minutes, the careful reports published in the *Philosophical Transactions* may give us an idea.^51^ Around 1670, Robert Boyle (1627–1691) had a gudgeon placed into a ‘Pneumatical Engin’, or air pump.^52^ The experiment, ‘far from being the first’ that had been done on a fish with this sort of instrument, was devised to show what happened to a fish when ‘it should be kept for some hours together from all supply of fresh Air’.^53^ Although after mostly all of the air was removed ‘there appeared a great store of Bubbles all about the Fish’, no definitive conclusions could be drawn.^54^ The *Historia piscium* lauds Boyle for his ‘most excellent experiments’ on the effects of water pressure upon bodies of air.^55^ It recounts an experiment to fill up a swim bladder with air and submerge it in a clear, deep vessel filled with water. The deeper the bladder was plunged, the more contracted it would become, and vice versa.^56^

Fellows did not only pursue their inquiries on fish within the confines of Gresham College, where their weekly meetings took place.^57^ Hooke recounts coming across a porpoise displayed at Ulbars (possibly a fishmonger) one day in November 1679.^58^ He bought the specimen and transported it to Garraway's coffee house, near the Royal Exchange.^59^ Here he performed a public dissection.^60^ Just like demonstrations of instruments, examinations of animal species in a tavern or coffee house could facilitate discourse on natural phenomena among individuals of various stripes.^61^ These might well be people possessing valuable experience, such as sailors. Hellawell has demonstrated, for example, how the Society considered seamen uniquely positioned to record and examine certain natural phenomena.^62^ While she proposes further case studies be conducted of the evaluation of the knowledge and skills of other occupational groups, she signals that this can be difficult, as such groups do not always fit ‘the conventional artisanal mold’.^63^ Like seamen, fishermen do not readily fall into those historiographical categories of workmen that have received sustained attention from historians of science over the past decades, notably invisible technicians and artisans. The work of fishermen and fishmongers was, after all, not technical in the sense that they handled (scientific) instruments—in contrast to, for example, those who assisted Boyle.^64^ They also do not quite resemble the self-aware artisans one might encounter in the works of Pamela Smith and Pamela Long, who created the texts and artefacts that have come down to us today, such as recipes, manuals, drawings, paintings, casts or ceramics.^65^

There is a lacuna of sources when it comes to fishermen and fishmongers. As the passage opening this section highlighted, the authors and compilers of the *Historia piscium* ultimately selected what was included in the book, and what was left out. Azzolini has argued that we ‘accord undue weight to the authority of writers’ when not taking the spoken word into account.^66^ Local and oral connections are indeed often overlooked as a result of the emphasis on texts when reconstructing early modern networks.^67^ The Royal Society archive contains one written trace of London fishmongers themselves: a petition they presented to Parliament, and which was read aloud at the Royal Society.^68^ They wished ‘that our Sea coste & rivers may swarme with the fry & brood of fish, & our Towns and Cittyes better provided for’ through stricter enforcement of the law prohibiting too many young fish from being taken.^69^ Besides offering a unique insight into these fishmongers' affairs, this document also reminds us that, while the relative inconspicuousness of fishermen and fishmongers may lead them to seem like a monolithic group, they had their own interests and backgrounds.^70^ It is nonetheless quite rare that fishermen and fishmongers are recognizable individuals, like the Strasbourg fisherman and burger Leonhard Baldner (1612–1694).^71^ His manuscript, *Vogel-, Fisch- und Thierbuch* (*Book of birds, fish and animals*), is cited throughout the *Historia piscium* and will be discussed in detail below. The remainder of this article sets out to reconstruct the nature, extent, diversity and significance of the contributions of practical men to the *Historia piscium*.

## Knowledge at the fish market

Fishermen take centre stage in the engraved title page of the *Historia piscium*, even if they are depicted as rather more gentile individuals than they probably were. Fishermen and fishmongers provided (if not always wittingly) the raw material for natural historical and philosophical investigations. When Willughby and Ray travelled through the British Isles and continental Europe, they frequented markets to get their hands on new species of birds and fish.^72^ As the latter described, they ‘visited almost all the chief fishing ports of England, and the markets of Belgium, Germany, Italy and France; … bought all the species new to us and described them so that the reader can easily recognize them’.^73^ Their daily visits to the fish market in Rome produced rich results, as ‘scarce any fish to be found anywhere on the coast of Italy but some time or other it may be met withal heer’.^74^ Ray's travel companion Philip Skippon (1600–1660) listed no fewer than 89 species of fish that they had come across at Venice's market.^75^ Visiting (fish) markets to spot new specimens was in fact a widely utilized practice. When stationed in Jamaica in the service of the Duke of Albemarle, for example, the physician and collector Hans Sloane (1660–1753) relied on local markets to access rare species.^76^

The piscine wealth to be found at fish markets was further proof that the underwater world teemed with creatures meriting closer examination. In one of his physico-theological treatises, Ray marvelled—echoing Psalm 104:25— ‘The Sea, what infinite Variety of Fish doth it nourish!’^77^ While fish were indeed wonderfully varied, Ray also believed that God had created a fixed number of species of them.^78^ It was a well-established tradition, after all, to consider the underwater realm as a divinely designed structure that mirrored the rational organization of the heavens.^79^ From the outset, the expectations for the *Historia piscium* were high. Ray wrote to the Royal Society: ‘For this history of fish, I can warrant it to be as full and perfect as to the number of species, and their descriptions … as was the history of birds.’^80^ Willughby and Ray's idea of a perfect fish book differed from those extensive volumes full of anecdotes, fables and proverbs that certain Renaissance authors compiled. Rather, they confined their study of natural creatures to ‘what properly relates to natural history’, as the latter put it, thereby excluding what they considered to be fabulous or folkloristic accounts.^81^ Their main issue with earlier authors, however, was that they had not been diligent enough in distinguishing one fish from the other, and so had caused an unnecessary duplication of species.^82^ As a solution to this muddle they defined clear ‘characteristic marks’ (*notae characteristicae*) that demarcated one species from another.^83^ These marks might be the number and position of its fins, certain spots or colours, or other properties. A tope shark, for example, could be discerned from the similar-looking smooth hound shark by its larger size, its rows of sharp teeth and its eyes, the irises of which were of a brighter, silver colour.^84^

Willughby and Ray thus aimed to uncover the ‘true’ (*viz.* God-given) arrangement of species by both establishing an unambiguous differentiation between species and seeking to understand how these were related to one another.^85^ Their study of fish, and of nature more generally, was carried out in the context of larger philosophical reflections on the connections between knowledge and language, an interest they shared with their fellow Royal Society member Bishop John Wilkins (1614–1672). Like sundry others at the time, he believed that God had confused people's tongues as a punishment for the arrogance they had displayed in building the Tower of Babel.^86^ Wilkins therefore set out to compose a universal language, by creating word tables that showed the true relation between words and things. Willughby and Ray both contributed to Wilkins' project, which eventually appeared as *An Essay Towards a Real Character, and a Philosophical Language* (London, 1668).^87^ Ray, however, would later privately admit to be ‘ashamed and disgusted’ to have been so publicly associated with a project that he found, at its core, to be ludicrous.^88^ While he agreed with the idea that a sound connection could—and should—be established between a word and a thing, he denounced the imposition of a pre-contrived system onto nature's rich variations. Rather, he was convinced that true knowledge came from the senses.^89^

When deploying the senses to study a species of fish, having recourse to a (more or less) fresh sample was much to be desired. Sometimes fishermen delivered specimens to the naturalist's doorstep. In a letter to the Royal Society detailing his dissection of a porpoise, Ray relates how, during his visit to Wilkins in Westchester in late April 1669, he had had ‘the good fortune to meet with a young porpess of a convenient size for dissection, brought thither by some fishermen, who caught him upon the sands, where the tide had left him’.^90^ These men seemed well aware that the novelty value of certain fish washed ashore could be converted into actual coin. Their hustling was rewarded; the bishop purchased the fish (for an unknown sum) and handed it to Ray for description.^91^ Dissecting animals was in fact a key component of Willughby and Ray's research. When, during their travels in Europe, they acquired a fresh specimen of fish or fowl, they often dissected it—or had this done for them by servants—to facilitate detailed and close observation.^92^ A fair amount of the species descriptions in the *Historia piscium* include detailed descriptions of internal organs. A set of four drawings in Willughby's archive record stages in the dissection of a male flair that took place under the supervision of Skippon.^93^ It is an exemplary piece of the kind of close observation that Willughby, Ray and their contemporaries held up as an ideal.

When no fresh specimen was at hand, they made do with preserved ones. Certain dried exemplars could, as we have learned, be sighted in taverns. The Royal Society itself also possessed a repository of objects. As the catalogue made for the Society by the natural historian and Fellow Nehemiah Grew (1641–1712) shows, the collection encompassed ‘humane rarities’, animals, plants and minerals.^94^ The subsection entitled ‘fish’ was devoted entirely to aquatic fauna.^95^ Although the collection was impressive, its value for making proper species descriptions was limited, because, as Michael Hunter has noted, ‘preserved exhibits were decidedly inferior to live ones’.^96^ The difference in utility between that of a living specimen and a dead, prepared one was especially marked in fish because they disintegrated so easily. Each method of preservation had its merits and pitfalls: submerging specimens in spirits, for example, was rather costly and not altogether attractive for display, whereas dried specimens could become brittle so that only the sturdier parts of the fish endured.^97^ Regardless of the preservation strategy used, fish often lost much, if not all, of their original colour. Images could address this problem—to an extent.

The importance of illustrations for the *Historia piscium* was signalled on its engraved title page by the inclusion of the helmeted artist. It also stated that any ‘new’ figures—that is, those that were not copied from earlier authors—had been marked with a dagger.^98^ These new figures were usually based on drawings that Willughby, Ray or others in their circle had acquired, and which were either sent to them by correspondents or purchased during travel or trip.^99^ The Society's committee also commissioned illustrations from specimens in its collection for inclusion in the work.^100^ The images selected were those that best conveyed the morphology of the fish.^101^ If drawn well, the species depicted could be determined. Another source for illustrations was a manuscript inscribed ‘A Book of Fishes done at Hamburgh, with Mr Ray's Notes’, that has hitherto received little attention from historians.^102^ It contains dozens of coloured illustrations of aquatic fauna, executed in watercolour and what appears to be gouache, accompanied by cursory descriptions in a German hand. Ray's annotations give insight into how this book was used. He comments, for example, on the correct identification of a species (‘these are not separate species, but the front and back side of the same fish’) or on the quality of certain drawings (‘badly painted’).^103^ While the natural historical value of illustrations was dependent on their being made from a fresh specimen by a skilled artist, whether or not this was the case could be difficult to ascertain if one had not personally seen a suitably lively, or at least fresh, example of the species. The qualifying phrase ‘drawn from the life’, multivalent in its early modern usages, can be said to take on special meaning in the case of fish.^104^

Meticulous attention to detail was highly desirable if fish were to be properly distinguished from one another, but Willughby and Ray disagreed on the subject of precisely how much of it was *needed*. Willughby's painstaking descriptions of the plumage of birds were met with some suspicion by Ray, as this oft-cited passage makes clear:

I must confess that in describing the colours of each single feather he [Willughby] sometimes seems to me to be too scrupulous and particular, partly because Nature doth not in all Individuals, (perhaps not in any two) observe exactly the same spots or strokes, partly because it is very difficult so to word descriptions of this sort as to render them intelligible.^105^

Besides addressing the limitations of language when it came to describing certain facets of species, like colour, Ray here exposes the problem of ascertaining whether a certain specimen was a distinct species or merely a variation within a species. Willughby and Ray often discuss this in their history of birds, but it also resurfaces in their fish book.^106^ As we will see, this is where the experiences of fishermen and fishmongers came in handy: they saw a relatively large quantity of each species of fish, and live examples at that, as opposed to the few dried exemplars available in natural historical collections, and thus had a larger ‘sample size’ of specimens from which they might draw conclusions.

**Figure 2.**
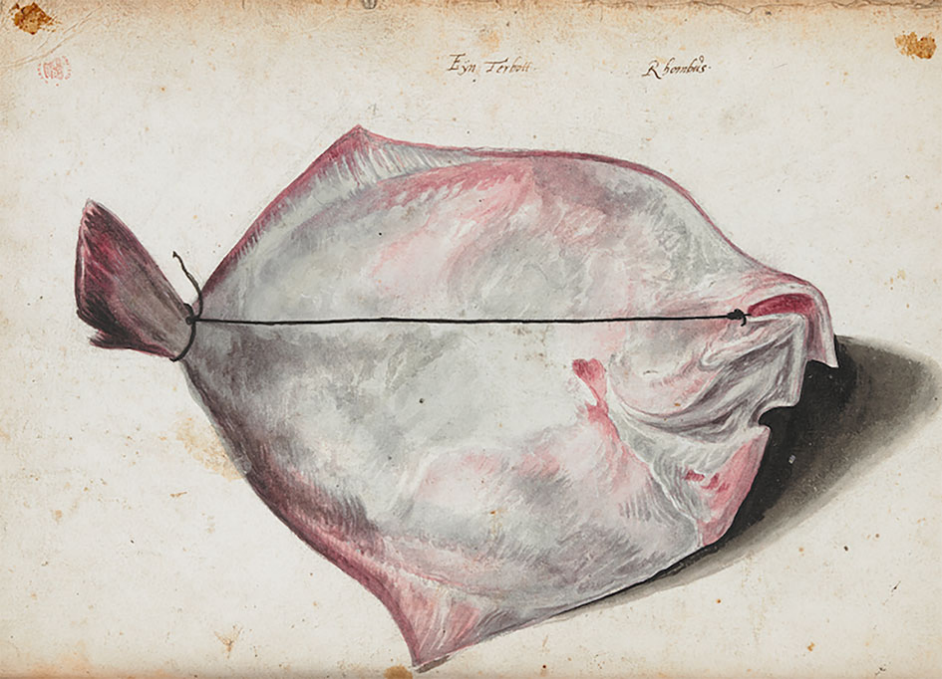
Drawing of a species of flatfish, inscribed ‘Eÿn Terbott’ and ‘Rhombus’ in unknown hand(s). (From Anon., ‘A Book of Fishes done at Hamburgh, with Mr Ray's Notes’, Sloane manuscripts, Add. MS 5308c, f. 4v, British Library, London. © British Library Board.) (Online version in colour.)

Before fish could be captured on paper, they first needed to be caught. One can easily forget this when looking at the engraved plates in the *Historia piscium*, which present the fish as if untouched by human hands, showing no sign of hooks or holes.^107^ An exception is the engraving of a species of flatfish that does convey obvious traces of capture: a thin black cord has been tied from its head to the peduncle of its tail.^108^ The engraving was based on one of the drawings (figure 2) in ‘A Book of Fishes done at Hamburgh, with Mr Ray's Notes’.^109^ This particular manner of tying up flatfish is depicted in various fish still lifes by seventeenth-century Netherlandish painters such as Abraham van Beijeren, Isaac van Duijnen and Jacob Foppens van Es. These still lifes often show fish specimens acted upon in one way or another: they are cut, sliced, smoked or tied. This way of binding a flatfish head to tail seems to have had very practical reasons, namely to facilitate its transport or delay the spoiling process.^110^ The illustration serves as a reminder that fish had to be caught, carried, stored and preserved before they could be subjected to scrutiny.

## Detail and distinction

Fishermen not only supplied the goods for natural historical research, but were themselves sources of embodied knowledge. They shared their know-how of fishing methods; on a par with the broader interest of the Fellows in the history of trades, species descriptions in the *Historia piscium* unfold the intricacies of catching herring or trapping tuna.^111^ They were mostly asked, however, about their knowledge of the occurrence of species. When Ray toured through the British Isles in 1662 with Willughby, he compiled catalogues of English birds, fish, metals and minerals.^112^ He noted down several fish taken around Penzance and St Ives in Cornwall, presented to him by ‘one of the ancientest and most experienced fishermen’, who remains nameless.^113^ Ray here stressed his informant's decades' worth of experience; other Fellows used similar phrasing while appealing to the seniority of the seamen whom they had consulted.^114^ The first entry on Ray's fish list was a whale, which the old fisherman had spotted from the coast. Ray added that he could not tell them of what sort it was, remarking that ‘*vulgus enim non distinguit*’—‘the common people, after all, do not distinguish’.^115^ In the *Historia piscium* it is similarly declared that fishermen do not really discern the mackerel from any other fish that may look like it.^116^ This seeming lack of interest in the categorization or classification of fish ran very much contrary to the earlier mentioned impetus of Ray and Willughby to precisely distinguish between species.

Ray's remark was somewhat unjust. Not only did the diversity to be found in fish present a complex puzzle, as species often closely resembled each other and could only be differentiated through subtle variation, but Ray actually drew on fishermen's own distinctions in trying to solve such conundrums. Consider the following passage, in which Willughby and Ray deliberate on whether sprats formed a separate species or were nothing more than the offspring of herring:

A certain senior fisherman from *Cornwall*, whom we have consulted about this matter and other things, has told us that two kinds of *Sprats* are caught in the sea which flows near to Cornwall, one of Herring, another of Pilchards or the offspring of Celerini, which can in turn easily be distinguished from another. Pilchards frequent the shores of Cornwall and Devon, they very rarely progress further to the east in the British sea; from whence elsewhere around England only one type of Sprat is found.^117^

Here, yet again, a fisherman—possibly that same wise and experienced individual—imparts his knowledge. His answers did not make matters simpler, as he explained that there are, in fact, different kinds of sprats, which stem from at least two different species, and that these are, furthermore, not equally distributed along the British coasts. A looming problem in these interactions was that a fish might have a different name in Cornwall from the one it would have in London. The ‘Scad’ in Cornwall was known as a ‘horse Mackrell’ in London; conversely, the species of flatfish that Londoners dubbed a ‘Pearle’, the Cornish called ‘Lug-aleaf’.^118^ In keeping with Willughby and Ray's preoccupations with language, the *Historia piscium* and its related writings abound with attempts to establish which fish went by which name where, and according to whom.

The taxonomies of fishermen did not always overlap with those of the naturalist. This added a linguistic layer to the already intricate puzzle presented by the various species. Ray wrote to Lister:

Of the flat cartilaginous [fish] I have seen and described four or five sorts, but I am to seek what our fishermen mean by the Skate [*Raia batis*], and what by Flair [Fireflaire, the Sting Ray, *Trygon pastinaca*], and what by Maid—as Skate-maid, Homelyn-maid, Thornback-maid, &c. &c.^119^

Distinctions between (or even within) species by people of practice also appear to have been based on attributes with particular relevance to their commerce. In the species description of the herring, it is explained that the people who washed, salted and dried this fish, and who were called Towers, separated it ‘into six species or rather grades’.^120^ These encompassed the ‘fat herring’, which was large and fat, and the ‘meat herring’, which was equally large and rich in meat but less fat.^121^ ‘Pluck’ was the name used for herring damaged or torn from being stuck in the nets, while a ‘shotten herring’ had emptied itself of its roe.^122^ We thus find, subsumed in Willughby and Ray's natural historical taxonomy based on characteristic marks, a taxonomy drawn up from properties stemming from commercial practice.

Ray's erstwhile fellow Cambridge student and the vicar of Brignall, Ralph Johnson (1629–1695), wrote him to complain of how difficult it was to decide whether dissimilar-looking exemplars of salmon were truly different species, or rather one and the same species in different stages of growth.^123^ He said that in

the mouth of Eden in Cumberland the fishers have four distinctions of yearly growth (after the first summer, when they call them free, or frie, as we smowts, or smelts) before they come to be lackes; and this, they say, they have curiously observed, by fixing so many pins in the fins of yearlings, or two years old, and after taking them again; …^124^

This method, of fixing pins into individual specimens and tracing their growth over a period of time, entailed an experiment. Like the experiments conducted by the Fellows, it was designed to draw out certain observations. Fishermen's distinctions between salmon of different ages were deemed dependable enough to be included into the book:

And what is handed down by authors about the quick *growth* of small salmon in the sea does not find faith with us: for our fishermen distinguish salmon by each year of their age, as we have said above, and they say that they are not full-grown before the sixth year of their life.^125^

Willughby and Ray thus trusted the collective account of ‘their’ fishermen over the written knowledge transmitted by various (here unspecified) earlier authors.

How could one tell whether a specimen was exemplary for its species? Fishermen and fishmongers had a good sense of irregularities and averages. Willughby and Ray were told by a fishmonger that bigger specimens of salmon weighed around 6 pounds.^126^ They also drew, albeit indirectly, on the observations of the Cambridge fishmonger Mr Mayfield, who went down to the London market every Friday to procure species not readily available in his own town.^127^ The physician Peter Dent wrote to Ray that ‘Mr. Mayfeild [*sic*] could not procure any dried *Mayds* or *Thornback* at the mart. He helped me to a fresh *Thornback*, which he said was full grown: its weight was ten pounds.’^128^ Dent added that the fishmonger was ‘acquainted with the Tamworth carrier and will undertake to send you any of these [fishes] fresh into the country’ and thus could also do deliveries.^129^ He had furthermore told Dent that he once sold an exceptionally large specimen of flair to the cook of St John's College, Cambridge, and it ended up feeding all those attending lunch that day. Dent sought verification of the story from the cook in question and, having received it, he passed it along to Ray, who then inserted it into the *Historia piscium*.^130^ The reader could rest assured that the fishmonger Mayfield was of trustworthy character (*fide dignus*).^131^

Fishermen and fishmongers could furthermore tell whether a certain specimen was male or female, and how particular species procreated. The dependable Mayfield, for example, assured Dent that flairs were viviparous.^132^ While Dent doubted whether this was true, he resolved to observe weekly the alterations of the fish's eggs and give Ray a full account.^133^ Although Dent's ultimate findings cannot be found in Ray's correspondence, the letter underscores that the statements of fishmongers, like those of fishermen, merited further research and that their claims invited both validation and repudiation.

The *Historia piscium* frequently cites the manuscript of Leonhard Baldner. He is the first fisherman in the book whose name we know; rarer still, his portrait has come down to us.^134^ Born into an established Strasbourg fishing family, whose crest consisted of three crossed fish, Baldner received an education, and combined his work as fisherman with a seat on the city council.^135^ He produced several, largely similar, manuscripts describing the birds, fish and other animals of his home region, most of which were skilfully illustrated by the painter Johann Georg Walther (1634–1697).^136^ Willughby bought one of these quarto volumes during his Continental tour.^137^ In the preface to the *Ornithology*, Ray expressed his appreciation of the high quality of the illustrations, praising their great exactness and excellent hand.^138^ It struck him that Baldner had taken and described these fish himself, and had them drawn at his own charge and cost. Such curiosity, Ray thought, was ‘much to be admired and commended in a Person of his Condition and Education’.^139^ He also acknowledged that he had received ‘much light and information from the Work of this poor man’, which had enabled him to ‘clear many difficulties, and rectifie some mistakes in *Gesner*’.^140^ Ray furthermore wrote to Robinson:

though it is not supposed, that a man of his education should be able to describe animals well, yet so much might be gathered from the notes he gives, as might lead an understanding and attentive man into the knowledge of them, and with the figures (which are in all very exact) give him so much light as to enable him to determine the species.^141^

**Figure 3.**
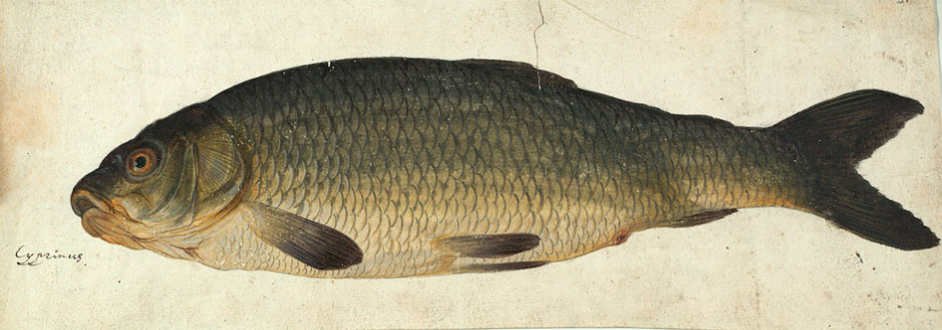
Watercolour of a species of carp, inscribed ‘Cyprinus’ in Willughby's hand. (From Nottingham University Library, Middleton Collection, Mi LM 25/51. © University of Nottingham Manuscripts Collections.) (Online version in colour.)

On the title page of his manuscript, Baldner proclaimed that both the species descriptions and the illustrations conformed to nature.^142^ Looking at a drawing that Willughby purchased from Baldner alongside the manuscript, a watercolour of a carp (figure 3), one can see why Ray was so enthused.^143^ The artist has drawn the fish from an ever so slight bottom perspective view, and diligently rendered the scales and fins; the latter, especially, show fine brushstrokes. By subtly applying a greyish, light blue paint to the edges of the gills and scales, a technique known as heightening, he has conveyed the glistening of a fish that has just been taken out of the water. The drawing was used for the *Historia piscium*.^144^ Baldner intended the descriptions and images in his manuscript to complement one another. He pointed out, for example, that, even though the species of ‘Rothaug’ closely resembled that of the ‘Rotel’, its colours were more beautiful, and its eyes and fins were more rubescent, as could be seen from the illustration.^145^ In their description of the ‘Rootaug’, Willughby and Ray used the same distinctive marks.^146^

The authors looked to Baldner's manuscript for a wider range of observations.^147^ They copied, for example, some of his statements on whether a certain species was rare or common in his area, how its appearance could vary with time or place, when and how it procreated, and when it was best to eat.^148^ To focus on only those parts of the manuscript included in the *Historia piscium*, however, is to miss out on many other fascinating observations. These include Baldner's account of having caught a sturgeon of ‘about the thickness of a man’, and subsequently finding its bowels to weigh 130 pounds.^149^ Thus, like Willughby, Ray and their peers, Baldner dissected fish and studied their internal anatomy; he even counted the thousands of eggs in the roe of pikes and turbots.^150^ He noticed that the species of wood trout took on the colours of their environment: they turned completely white when placed in a white tub, and black once put in a black tub.^151^ He disagreed with Gessner that carp were (sometimes) born from mud, and said that they all came from roe.^152^ All in all, Baldner's manuscript demonstrates that he aimed to distinguish species from one another, to examine their anatomies and to understand how they behaved and procreated, and that he held his own observations against those described by earlier authors—again, much like Willughby and Ray.

The introduction to Baldner's manuscript (dated 31 December 1653) gives us a sense of how he envisioned his work. It reveals that the author thought there to be no better place to contemplate God's omnipotence than on and near the water. Since God had at the beginning created the great whales, fish had received his first blessing; and he had also called upon the fishermen to follow him. God had, furthermore, made the rivers of the Rhineland with their endless benefits to those who lived around them. It was this delight in and admiration for the creation, Baldner submitted, that had inspired him to make this manuscript brimming with animals that swam, flew and crept in these waters. He wrote that all of the creatures described in it he had held in his own hands. Each of the species was drawn from life, called by its name and, after sustained study, described briefly from Baldner's own ‘experience’ (*Erfahrung*).^153^ He admitted that his attempts were necessarily ‘simple’ (*einfältig*) and ‘scant’ (*gering*), casting himself as a modest fisherman and hunter, and bade those considering themselves better suited to write such a work to keep that humble background in mind.^154^ At the same time, he emphasized his three decades' worth of experience with fish—although he used the words ‘learned’ (*erlernt*) and ‘studied’ (*studiert*).^155^ Quite apart from its complicating of certain assumptions about what constitutes ‘the’ fisherman, Baldner's manuscript also testifies to the fluid boundaries of theoretical and practical engagement with nature.

## Conclusion

Let us return to the salmon, and its curious behaviours, one last time. A few lines after its peculiar matter of jumping is discussed, its mysterious eating habits are addressed: ‘What food salmons use, because I see that authors disagree [on the matter], has to be consulted by experience.’^156^ This matter had been discussed at a meeting of the Royal Society in 1678, where it was brought forth that fishmongers never found anything in the maws of salmon and that an (unnamed) lady, ‘very inquisitive in that kind’, had observed the same.^157^ The previous year, Johnson had written to Ray: ‘I wonder as much that Fishers have not certainly determined whether Salmons live upon anything save Water, and what?’^158^ He continued by noting that

I think only the Anglers have made the Observation of finding their Stomachs always empty; but I am persuaded that, if the Net-fishers would open any considerable Number, they would find in them Food indigested, which they seldom do, but sell them whole. Perhaps I may give farther Answer to this *Quaere*, and some others about *Whitsontide*; at which Time I purpose to go to our Coasts, and gather what I can.^159^

These discourses are indicative of the sorts of questions over which naturalists pondered, and where they expected to find answers.

In recent years, historians have widened their conceptions of the spatial range in which natural knowledge was created, and shown how this should be approached.^160^ For London, fertile sites for assembling knowledge about fish encompassed—besides the rooms of Gresham College—coffeehouses, taverns, ports, fishmongers and the banks of the Thames.^161^ Beyond the confines of the city, such locations included the coast of Cornwall and (fish) markets in continental Europe. Each of these places allowed the making of first-hand observations, but, even more pressingly, meeting those people whose observations of fish were informed by years of practice. This article has tried to reconstruct the conversations between fishmongers and fishermen and Fellows so as to better comprehend what they actually entailed, and to analyse how these contributed to a deepened understanding of fish. It has also emphasized how the extent and nature of these contributions might differ from person to person, relative to experience and skill. Taken together, the various examples discussed here demonstrate that exchanges with men of practice were not incidental, but rather central to Willughby and Ray's project.

This is not to say that interactions between practical men and Fellows could not also be rather complicated. As this article has shown, fishermen and Fellows sometimes talked at cross-purposes, reminding us of similar difficulties in communication that arose in the Society's history of trades project.^162^ Another issue was that, while the Fellows appropriated knowledge from people of practice for their discussions or publications, the practitioners themselves were often made to disappear from sight.^163^ This also held true for others, not discussed in this article, whose observations of fish were drawn upon for the *Historia piscium* and which merit further consideration. Anglers, too, knew their way around fish. Willughby and Ray consulted Leonard Mascall's well-known angling manual, *A Booke of Fishing with Hooke & Line, and of All Other Instruments There-unto Belonging* (London, 1590), when discussing the fact that, while the carp was a relatively recent introduction to the waterways of England, it was now plentiful in rivers and ponds.^164^ Anglers were also aware of whether a species was common or rare and, as Johnson implied, knew what was in a fish's stomach. Other specific knowledge of fish pertains to their consumption. As we saw, *Historia piscium* offers glimpses of fish salters and cooks; furthermore, the taste and preparation of fish species receives occasional attention in the book.^165^

The *Historia piscium* was an attempt to create a universal work on the natural history of fish based on clearly defined principles, so that the proper relations between species and their names could be re-established, and order restored in the wonderfully varied world of fish. The many sources on which Willughby, Ray and other Fellows of the Royal Society drew reflect some of this variety. The value of interacting with fishermen and fishmongers lay in their repeated engagement with a large quantity and wide variety of fresh fish in an either living or recently deceased state. They not only supplied raw material, but also offered information that was crucial for the central tenet of the *Historia piscium*: to distinguish one species from another and delineate their differences. Fishermen and fishmongers not only knew how to catch fish and how to tell them apart from another, but also commented on particular behaviours of certain species. The interactions between fishermen and fishmongers and the learned world with regard to classification and categorization, as discussed here, continued well into the nineteenth and twentieth centuries, and still pertain.^166^

## Footnotes

1 Francis Willughby and John Ray, *Historia piscium libri quatuor* (Sheldonian Theatre, Oxford, 1686) (henceforth *Hist. pisc.*), RCN 18574, Library and Archives of the Royal Society, London. The annotations are the remarks of Tancred Robinson (TB) penned down by Francis Aston; later annotations are Cromwell Mortimer's (CM).

2 *Ibid.*, pp. 96–97, 105 (TB).

3 *Ibid.*, p. 28 (CM).

4 *Ibid.*, p. 166 (CM).

5 Thomas Birch, *The History of the Royal Society for Improving of Natural Knowledge*, 4 vols (A. Millar, London, 1756–1757), vol. 4, p. 42.

6 This elusiveness is explored in Elspeth Graham, ‘Ways of being, ways of knowing: fish, fishing, and forms of identity in seventeenth-century English culture’, in *Animals and early modern identity* (ed. Pia F. Cuneo), pp. 351–373 (Ashgate, Farnham, 2014). The phrase ‘slippery denizens’ comes from Matthew C. Hunter, *Wicked intelligence: visual art and the science of experiment in Restoration London* (University of Chicago Press, Chicago, 2013), p. 69.

7 Brian Ogilvie, *The science of describing: natural history in Renaissance Europe* (University of Chicago Press, Chicago, 2008), p. 259.

8 The book was originally envisioned as two separate works; one work containing texts, and the other (to bear the name *Icthyographia*) illustrations. While the works were published together as one book, each retains its own title page. See Adrian Johns, *The nature of the book: print and knowledge in the making* (University of Chicago Press, Chicago, 2000), p. 489.

9 A print proof of the frontispiece, without title and affiliation engraved in, can be found in Francis Willughby's archive in the Middleton Collection, Manuscripts and Special Collections, University of Nottingham (henceforth Middleton Collection). The illustration is part of his notebooks encompassing illustrations of, *inter alia*, birds, fishes, insects and flowers (shelfmark Mi LM 24/170).

10 Anna Marie Roos has suggested that the ship on the title page is a visual nod to the one displayed on Bacon's *Instauratio magna* (John Bill, London, 1620). See Anna Marie Roos, *Web of nature: Martin Lister (1639–1712), the first arachnologist* (Brill, Leiden, 2011), p. 325.

11 For a discussion of title pages of natural historical works on fish, see Paul J. Smith and Didi van Trijp, ‘Dynamiques européennes de l'humanisme érudit dans l'histoire naturelle: le cas de l'ichtyologie, de Belon, Rondelet et Gessner à Willughby et Ray’, in *L'humanisme à l'épreuve de l'Europe (XVe–XVIe siècles)* (ed. Denis Crouzet, Elisabeth Crouzet-Pavan, Philippe Desan and Clémence Revest), pp. 167–181 (Champ Vallon, Ceyzérieu, 2019).

12 Volker R. Remmert, ‘“Docet parva picture, quod multae scripturae non dicunt”: frontispieces, their functions, and their audiences in seventeenth-century mathematical sciences’, in *Transmitting knowledge: words, images, and instruments in early modern Europe* (ed. Sachiko Kusukawa and Ian Maclean), pp. 239–270 (Oxford University Press, Oxford, 2006), at p. 240.

13 For a detailed study of the sources for the book, see Sachiko Kusukawa, ‘*Historia piscium* (1686) and its sources’, in *Virtuoso by nature: the scientific worlds of Francis Willughby (FRS*) (ed. Tim R. Birkhead), pp. 305–334 (Brill, Leiden, 2016).

14 Other studies of the *Historia piscium* can be found in Kusukawa, *op. cit.* (note 13); Sachiko Kusukawa, ‘The *Historia piscium* (1686)’, *Notes Rec. R. Soc. Lond.* **54**, 179–197 (2000); and Charles E. Raven, *John Ray, naturalist: his life and works* (Cambridge University Press, 1942), esp. pp. 339–370.

15 It was indeed often men; no fishwives figure in the sources examined here. In England, fishwives were not allowed to sell inside public marketplaces: see Alena Buis, Christi Spain-Savage and Myra E. Wright, ‘Attending to fishwives: views from seventeenth-century London and Amsterdam’, in *Mapping gendered routes and spaces in the early modern world* (ed. Merry E. Wiesner-Hanks), pp. 177–200 (Ashgate, Farnham, 2015), at p. 193.

16 Original Latin: ‘Salmo adversus fluvios perpetuo nititur, cumque in ascensu sepem vel aliud hujusmodi obstaculum invenerit, in circulum flexo corpore caudam ore apprehendit, eamque mordicus tenens, iterumque dimittens magno impetu transilit. *Author de natura rerum* apud *Gesner*. Hoc à plurimis piscatoribus assidue fieri multoties audivimus. Quod Salmones ad saliendum agillimi sunt, libenter concedimus, & experientia quotidiana confirmat: verum quod de caudae apprehensione fertur minus verisimile nobis videtur.’ *Hist. pisc.*, p. 192.

17 Conrad Gessner, *Historia animalium liber IV* (Christopher Froschauer, Zurich, 1558), p. 974. This part is devoted to fish and other aquatic animals.

18 For example, Pierre Belon, Hippolyto Salviani, Ulysse Aldrovandi and Guillaume Rondelet. On the last, see Gillian Lewis, ‘The debt of John Ray and Martin Lister to Guillaume Rondelet of Montpellier’, *Notes Rec. R. Soc. Lond.* **66**, 323–339 (2012).

19 This debate is addressed in Isabelle Charmantier, Dorothy Johnston and Paul J. Smith, ‘The legacies of Francis Willughby’ in Birkhead, *op. cit.* (note 13), pp. 360–385, at pp. 382–385.

20 Dorothy Johnston, ‘The life and domestic context of Francis Willughby’, in Birkhead, *op. cit*. (note 13), pp. 1–43, at pp. 1–2.

21 Kusukawa, *op. cit.* (note 14), p. 187; for Lister's contributions, see Roos, *op. cit.* (note 10), pp. 318–332.

22 For example, the experiment for determining the centres of gravity for a pilchard and a herring by holding the specimens by the tip of their back-fin: Tancred Robinson, letter to John Ray, 8 September 1685, in Edwin Lankester (ed.), *The correspondence of John Ray* (The Ray Society, London, 1848), p. 174; see also *Hist. pisc.*, p. 224.

23 Kusukawa, *op. cit.* (note 14), pp. 179, 187.

24 Not to the exclusion of engagement with texts: see Fabian Krämer, *Ein Zentaur in London. Lektüre und Beobachtung in der frühneuzeitlichen Naturforschung* (Didymos Verlag, Korb, 2014), p. 380.

25 Original Latin: ‘duntaxat tradere quae vel nobismetipsis & amicis observata essent, vel idoneos & fide dignos testes & auctores haberent’. *Hist. pisc.*, appendix, p. 30.

26 See, for example, Alberto Vanzo (ed.), *Experience in natural philosophy and medicine*, special issue of *Perspectives Sci.* **24**, 255–379 (2016); Ogilvie, *op. cit.* (7), pp. 17–23.

27 Peter Dear, *Discipline and experience: the mathematical way in the Scientific Revolution* (University of Chicago Press, Chicago, 1995), p. 109. See also ‘*experientia constat*’, *Hist. pisc.* p. 7; ‘*experientia didicimus*’, *ibid*., pp. 9, 16; ‘*id quod experientia mihi confirmavit*’, *ibid.*, p. 246.

28 Gianna Pomata, ‘Observation rising: birth of an epistemic genre, ca. 1500–1650’, in *Histories of scientific observation* (ed. Lorraine Daston and Elizabeth Lunbeck), pp. 45–80 (University of Chicago Press, Chicago, 2011).

29 While it is impossible to do justice to the intricacies of Bacon's epistemology or historiography here, I mention particularly on natural history Guido Giglioni, Dana Jalobeanu and Sorana Corneanu (eds), *Francis Bacon and the reconfiguration of early modern natural history*, special issue of *Early Sci. Med.* **17**, 1–271 (2012).

30 Guido Giglioni, ‘Learning to read nature: Francis Bacon's notion of experiential literacy (*Experientia Literata*)’, *Early Sci. Med.* **18**, 405–434 (2013), at p. 409.

31 Francis Bacon, *The Instauratio magna part II: Novum organum and associated texts* (ed. Graham Rees and Maria Wakely) (Oxford University Press, Oxford, 2004), p. 451.

32 *Ibid*., pp. 16–17.

33 Dana Jalobeanu, ‘Disciplining experience: Francis Bacon's experimental series and the art of experimenting’, *Perspectives Sci.* **24**, 324–342 (2016), at p. 336.

34 Deborah E. Harkness, *The jewel house: Elizabethan London and the Scientific Revolution* (Yale University Press, New Haven, 2007), p. 213.

35 Florike Egmond, ‘On northern shores: sixteenth-century observations of fish and seabirds (North Sea and North Atlantic)’ in *Naturalists in the field: collecting, recording and preserving the natural world from the fifteenth to the twenty-first century* (ed. Arthur MacGregor), pp. 129–148 (Brill, Leiden, 2018), at p. 131.

36 Anthony Grafton, ‘Philological and artisanal knowledge making in Renaissance natural history: a study in cultures of knowledge’, *Hist. Humanities* **3**, 39–55 (2018), at pp. 43–45.

37 Monica Azzolini, ‘Talking of animals: whales, ambergris, and the circulation of knowledge in seventeenth-century Rome’, *Ren. Stud.* **31**, 285–318 (2017), at p. 318.

38 Steven Shapin, *A social history of truth: civility and science in seventeenth-century England* (University of Chicago Press, Chicago, 1994), pp. 122–123.

39 Kusukawa, *op. cit.* (note 13), p. 333.

40 Steven Shapin and Simon Schaffer, *Leviathan and the air-pump: Hobbes, Boyle and the experimental life* (Princeton University Press, Princeton, 1985), p. 58.

41 Barbara Shapiro, *A culture of fact: England 1550–1720* (Cornell University Press, Ithaca, 2000), p. 140.

42 Peter Dear, ‘Totius in verba: rhetoric and authority in the early Royal Society’, *Isis* **76**, 144–161 (1985), at p. 156.

43 Philippa Hellawell, ‘“The best and most practical philosophers”: seamen and the authority of experience in early modern science’, *Hist. Sci.* **58**, 28–50 (2019), at p. 32.

44 Felicity Henderson, ‘Robert Hooke and the visual world of the Royal Society’, *Perspectives Sci.* **27**, 395–434 (2019), at p. 397.

45 Steven Pumfrey, ‘Who did the work? Experimental philosophers and public demonstrators in Augustan England’, *Brit. J. Hist. Sci.* **28**, 131–156 (1995), at p. 153.

46 Bacon, *op. cit.* (note 31), p. 131. It is important to note that he did not apply the notions *experientia*, *experimentum* and *observatio* too strictly: see Lorraine Daston, ‘The empire of observation, 1600–1800’, in Daston and Lunbeck *op. cit.* (note 28) pp. 81–113, at p. 83.

47 Peter Anstey, ‘Philosophy of experiment in early modern England: the case of Bacon, Boyle and Hooke’, *Early Sci. Med.* **19**, 103–132 (2014); Michael Hunter, ‘Robert Boyle and the early Royal Society: a reciprocal exchange in the making of Baconian science’, *Brit. J. Hist. Sci.* **40**, 1–23 (2007).

48 For example, 25 June 1662, JBO/1/66; 15 April 1663, JBO/1/159; 30 December 1663, JBO/2/23; all in Library and Archives of the Royal Society, London. Richard Shortgrave (d. 1676) may have been the Operator: see Marie Boas Hall, *Promoting experimental learning: experiment and the Royal Society, 1660–1727* (Cambridge University Press, Cambridge, 1991), p. 27.

49 Entry of 18 June 1662, JBO/1/66, Library and Archives of the Royal Society, London.

50 Entry of 24 June 1663, JBO/1/194, Library and Archives of the Royal Society, London.

51 For a discussion of the early *Philosophical Transactions*, see Adrian Johns, ‘Miscellaneous methods: authors, societies and journals in early modern England’, *Brit. J. Hist. Sci.* **33**, 159–186 (2000), at pp. 165–174.

52 Robert Boyle, ‘New pneumatical experiments about respiration’, *Phil. Trans. R. Soc. Lond.* **5**, 2011–2031 (1670), at p. 2011.

53 *Ibid*., p. 2024.

54 *Ibid.*, p. 2025. The specimen lived for some 10 days more; Boyle's postscript that ‘divers Gudgeons since taken dy'd there in much fewer dayes’ (*ibid.*, p. 2026) suggests that several trials were run.

55 *Hist. pisc.*, p. 8.

56 Similar questions are asked in A. I. and Robert Boyle, ‘A conjecture concerning the bladders of air that are found in fishes, communicated by A.I.; and illustrated by an experiment suggested by the Honorable Robert Boyle’, *Phil. Trans. R. Soc. Lond.* **10**, 310–311 (1675). The experiment entailed placing a specimen into a tall, long-necked vessel filled with water, and observing whether upward or downward motions of the fish caused changes in the water level.

57 Michael Hunter, ‘A “college” for the Royal Society: the abortive plan of 1667–1668’, *Notes Rec. R. Soc. Lond.* **38**, 159–186 (1984), at p. 159.

58 Robert Hooke, *The diary of Robert Hooke, 1672–1680* (ed. Henry W. Robinson and Walter Adams) (Taylor and Francis, London, 1935), pp. 430–431. See also Hunter, *op. cit.* (note 6), p. 118.

59 Rob Iliffe, ‘Material doubts: Hooke, artisan culture and the exchange of information in 1670s London’, *Brit. J. Hist. Sci.* **28**, 285–318 (1995), at p. 286.

60 See Noah Moxham, ‘Edward Tyson's *Phocaena*: a case study in the institutional context of scientific publishing’, *Notes Rec. R. Soc. Lond.* **66**, 235–252 (2012).

61 Adrian Johns, ‘Coffeehouses and print shops’, in *The Cambridge history of science* (ed. Katherine Park and Lorraine Daston), pp. 320–340 (Cambridge University Press, Cambridge, 2006), at p. 336.

62 The Fellows asked them to conduct experiments and make observations while at sea, for example recording species of birds and fish, and magnetic variations of the tides. Hellawell, *op. cit.* (note 43), pp. 36, 46.

63 *Ibid*., pp. 33–34.

64 Steven Shapin, ‘The invisible technician’, *Am. Scientist* **77**, 544–563 (1989); Rob Iliffe (ed.), *Technicians*, special issue of *Notes Rec. R. Soc. Lond.* **62**, 3–148 (2008).

65 Pamela O. Long, *Artisan/practitioners and the rise of the new sciences, 1400–1600* (Oregon State University Press, Corvallis, OR, 2011); Pamela Smith, *The body of the artisan: art and experience in the Scientific Revolution* (University of Chicago Press, Chicago, 2004).

66 Azzolini, *op. cit.* (note 37), pp. 299–301.

67 Ruth Ahnert, ‘Maps versus networks’, in *News networks in early modern Europe* (ed. Joad Raymond and Noah Moxham), pp. 130–157 (Brill, Leiden, 2016), at pp. 131, 140.

68 Cl.P.15i/8, Classified Papers, Library and Archives of the Royal Society, London. It was read on 23 September 1663 and brought in by John Graunt (1620–1674).

69 *Ibid*.

70 An overview of the various types of fishing in different European regions can be found in A. R. Michell, ‘The European fisheries in early modern history’, in *The Cambridge economic history of Europe* (ed. E. E. Rich and C. H Wilson), pp. 133–184 (Cambridge University Press, Cambridge, 1977).

71 An exception for the sixteenth century is Adriaen Coenen (1514–1587), author of the manuscript entitled *Visboeck* (*Fish book*). He is discussed in Egmond, *op. cit.* (note 35), pp. 132–139.

72 These travels are chronicled in John Ray, *Observations Topographical, Moral and Physiological, Made in a Journey through Part of the Low-Countries, Germany, Italy and France* (John Martyn, London, 1673); and Philip Skippon, ‘An Account of a Journey Made Thro. Part of the Low Countries, Germany, Italy and France’, in *A Collection of Voyages and Travels* (ed. Awnsham Churchill and John Churchill), 6 vols, vol. 6, pp. 359–736 (Messrs Churchill, London, 1732). See also Mark Greengrass, Daisy Hildyard, Christopher D. Preston and Paul J. Smith, ‘Science on the move: Francis Willughby's expeditions’, in Birkhead, *op. cit.* (note 13), pp. 142–226.

73 As quoted in Raven, *op cit.* (note 14), p. 365.

74 Ray, *op. cit.* (note 72), p. 362.

75 Skippon, *op. cit.* (note 72), p. 496; Kusukawa, *op. cit.* (note 13), p. 323.

76 James Delbourgo, *Collecting the world: the life and curiosity of Hans Sloane* (Allen Lane, London, 2017), p. 91.

77 John Ray, *The Wisdom of God Manifested in the Works of the Creation* (W. Innys, London, 1691), p. 78.

78 Kusukawa, *op. cit.* (note 13), p. 313.

79 James Delbourgo, ‘Divers things: collecting the world underwater’, *Hist. Sci.* **49**, 149–185 (2011), at p. 155.

80 John Ray, letter to Tancred Robinson, 13 March 1684, in Lankester, *op. cit.* (note 22), p. 164.

81 Tim R. Birkhead, Paul J. Smith, Meghan Doherty and Isabelle Charmantier, ‘Willughby's ornithology’, in Birkhead *op. cit.* (note 13), pp. 268–304, at p. 268.

82 Kusukawa, *op. cit.* (note 14), pp. 182–184.

83 Kusukawa, *op. cit.* (note 13), p. 308.

84 See the descriptions of ‘Mustelus laevis secundus’ and ‘Mustelus laevis primus’ in *Hist. pisc.*, p. 51 and p. 60 respectively.

85 Birkhead *et al.*, *op. cit.* (note 81), p. 269.

86 Kusukawa, *op. cit.* (note 14), p. 183.

87 Ray worked on the plants, Willughby on the animals. John Wilkins, *An Essay Towards a Real Character, and a Philosophical Language* (S. Gellibrand and John Martyn, London, 1668).

88 David Cram, ‘Francis Willughby and John Ray on words and things’, in Birkhead, *op. cit.* (note 13), pp. 244–267, at p. 255.

89 Kusukawa, *op. cit.* (note 14), p. 184.

90 John Ray, ‘An account of the dissection of a Porpess, promised numb. 74; made, and communicated in a letter of Sept. 12 1671, by the learned Mr. John Ray, having there in obser'd some things omitted by Rondeletius’, *Phil. Trans. R. Soc. Lond.* **76**, 2274–2279 (1671), at p. 2274.

91 *Hist. pisc.*, p. 32.

92 Kusukawa*, op. cit.* (note 13), p. 316.

93 Middleton Collection, Mi LM/12–15; 14 and 15 are reproduced in Tim R. Birkhead, *The wonderful Mr. Willughby: the first true ornithologist* (Bloomsbury, London, 2018), p. 120.

94 Nehemiah Grew, *Musaeum Regalis Societatis, or, A catalogue & description of the natural and artificial rarities belonging to the Royal Society and preserved at Gresham Colledge* (W. Rawlins, London, 1681).

95 It included the ‘rib of a triton, or mareman’, whale bones, seals, the claw of a lobster and the heads of manatees and dolphins—attesting to the wide category that ‘fish’ could cover in this period. Grew, *op. cit.* (note 94), pp. 81–103.

96 Michael Hunter, *Establishing the new science: the experience of the early Royal Society* (Boydell Press, Suffolk, 1989), p. 152.

97 Peter Davis, ‘Collecting and preserving fish: a historical perspective’, in MacGregor, *op. cit.* (note 35), pp. 149–165; Marlise Rijks, ‘Scales, skins, and carapaces in Antwerp collections’, in *The matter of mimesis: studies on mimesis and materials in nature, art and science* (ed. M. Bol and E. Spary) (Brill, Leiden, forthcoming).

98 Original Latin: ‘Figurae Novae, quae non paucae sunt, pugione notantur’; the daggers have not, however, been consistently applied: see Kusukawa, *op. cit.* (note 14), p. 186.

99 Kusukawa, *op. cit.* (note 13), pp. 307, 318–332.

100 Engravings of 12 collection specimens were incorporated into the *Historia piscium*, inscribed M.R.S. for Musaeum Regalis Societatis.

101 Roos, *op. cit.* (note 10), p. 322.

102 Anon., ‘A Book of Fishes done at Hamburgh, with Mr Ray's Notes’, Sloane manuscripts Add. MS 5308c, British Library, London; I thank Sachiko Kusukawa for drawing my attention to this source.

103 Original Latin: ‘Non sunt distincta species, sed ejusdem piscis pars supina et prona’ (Anon., *op. cit.* (note 102), f. 2v); ‘male pingitur’ (*ibid.*, f. 5v).

104 See Sachiko Kusukawa, ‘Ad vivum images and knowledge of nature in early modern Europe’, in *Ad vivum? Visual materials and the vocabulary of life-likeness in Europe before 1800* (ed. Thomas Balfe, Joanna Woodall and Claus Zittel), pp. 89–121 (Brill, Leiden, 2019).

105 Birkhead *et al.*, *op. cit.* (note 81), pp. 269–270.

106 *Ibid*., p. 275.

107 Compare the drawing of a spiky blowfish that Gessner had drawn, including a hook and tasselled string, with its printed counterpart in Gessner, *op. cit.* (note 17), p. 155, where these have not been represented, although a trace is still visible through a slight bump on the body. See Florike Egmond, *Eye for detail: images of plants and animals in art and science 1500–1630* (Reaktion Books, London, 2017), pp. 160–163.

108 *Hist. pisc.*, table F1.

109 Anon., *op. cit.* (note 102), f. 4v.

110 Julie Berger Hochstrasser, ‘From the waters: fish still life’, in *The magic of things: still-life painting, 1500–1800* (ed. Jochen Sander), pp. 185–211 (Hatje Cantz, Berlin, 2008), at p. 188; Anon., ‘Description of Isaac van Duynen's “Stilleven met vissen op een tafel”’, *Hoogsteder J.* **3**, 21 (1997).

111 *Hist. pisc.*, pp. 220 and 178.

112 John Ray, *A Collection of English words, not generally used … in two alphabetical catalogues, … northern … [and] southern counties, with catalogues of English birds and fish, and an account of preparing … metals and minerals* (Thomas Burrell, London, 1674).

113 *Ibid.*, p. 97.

114 Hellawell, *op. cit.* (note 43), p. 44.

115 Ray, *op. cit.* (note 112), p. 97.

116 *Hist. pisc.*, p. 182.

117 Original Latin: ‘Piscator quidam senior *Cornubiensis*, quem super hac re aliisque consuluimus, nobis retulit duo *Sprattorum* genera in mari Cornubiam alluente capi, alterum Harengorum, alterum Pilcardorum, seu Celerinorum sobolem, quae à se invicem facile distingui possint. Pilcardi Cornubiae & Devoniae littora frequentant, ulterius in mari Britannico orientem versus raro progrediuntur; unde alibi circa Angliam unicum tantum Sprattorum genus invenitur.’ *Hist. pisc.*, p. 221.

118 *Hist. pisc.*, pp. 95, 290; handwritten addition to species description in Willughby and Ray, *op. cit.* (note 1), table F1.

119 John Ray, letter to Martin Lister, 19 December 1674, in Lankester, *op. cit.* (note 22), p. 113.

120 Original Latin: ‘In sex autem species seu potius ordines …’; *Hist. pisc.*, p. 220. I thank Hans Aili for sharing his translation of this passage.

121 *Ibid*.

122 *Ibid*.

123 Johnson also shared observations on and specimens of birds and plants. Raven, *op. cit.* (note 14), pp. 249, 319. A biographical note can be found in *Teesdale Record Society* **15**, 9–32 (1945).

124 Ralph Johnson, letter to John Ray, 16 April 1677, in Lankester, *op. cit.* (note 22), p. 127.

125 Original Latin: ‘Quae de celeri Salmunculorum in mari *auctu* ab Autoribus traduntur apud nos fidem non inveniunt: nostratis enim piscatores Salmones annuatim ab aetate distinguunt, ut superius diximus, neque ante sextum aetatis annum perfici aiunt.’ *Hist pisc.*, p. 192.

126 *Hist. pisc.*, p. 196.

127 Raven, *op. cit.* (note 14), p. 393.

128 Peter Dent, letter to John Ray, 15 February 1674, in Lankester, *op. cit.* (note 22), pp. 15–17.

129 Peter Dent, letter to John Ray, 15 February 1674, passage omitted in Lankester, but reproduced in Robert W. Theodore Gunther, *The further correspondence of Ray* (The Ray Society, London, 1928), p. 113.

130 Where it was now claimed that the flair had fed all of the College's 120 alumni. Kusukawa, *op. cit.* (note 13), p. 331; and *Hist. pisc.*, p. 69.

131 *Hist. pisc*., p. 69.

132 Peter Dent, letter to John Ray, undated, in Lankester, *op. cit.* (note 22), p. 120.

133 *Ibid*.

134 The portraits are at Nottingham University Library (Middleton Collection, Mi LM25/80) and Brown University. See Kusukawa, *op. cit.* (note 13), p. 320.

135 Hans-R. Fluck and Albert Scharbach, ‘Leonhard Baldner: zu seinem Testament and Nachlassverzeichniss’, *Rev. Alsace* **142**, 283–297 (2016), at p. 293. He also collected duties on the Rhine: see Armin Geus, ‘Leonhard Baldner, a Strasbourg fisherman’, *Isis* **55**, 195–199 (1964), at p. 196.

136 The copies are discussed in Birkhead, *op. cit.* (note 93), pp. 101–103.

137 Leonhard Baldner, *Vogel-, Fisch- und Thierbuch* (*Book of Birds, Fish and Animals*), Add. MS 6485, British Library, London.

138 Francis Willughby and John Ray, *The Ornithology of Francis Willughby* (John Martyn, London, 1678), p. vi.

139 *Ibid*.

140 *Ibid*. Ray did not read German, and used Frederick Slare's abridged translations of the species descriptions, Add. MS 6486, British Library, London, ff. 12r–23v. That Ray also engaged directly with Baldner's manuscript is demonstrated by the Latin names he added to some of its descriptions.

141 Birch, *op. cit.* (note 5), vol. 4, p. 390.

142 Original German: ‘Recht Naturliche Beschreibung Und abmahlung’ (Baldner, *op. cit.* (note 137), f. 1r); inserting the word ‘recht’, Baldner modestly says they are ‘almost’ natural.

143 Middleton Collection, Mi LM 25/51.

144 *Hist. pisc.*, table Q1. Of the 40 drawings in Baldner, 25 were used. The other loose drawings are a perch (Middleton Collection, Mi LM 25/58) and a portrait (Middleton Collection, Mi LM 25/80). The former is represented in Kusukawa, *op. cit.* (note 13), pp. 320–321.

145 Original German: ‘Die Rothaugen sehen den Rottlen nicht ohngleich, seind aber von farben hüpscher, und Rothere Augen, und Schwümfedern, wie von dem abgemahlten zu sehen’ (Baldner, *op. cit.* (note 137), f. 135v.

146 *Hist. pisc.*, p. 249. Some confusion around the identification of this species is related in Birch, *op. cit.* (note 5)*,* vol. 4, p. 390.

147 *Hist. pisc.*, preface.

148 Baldner's manuscript is referenced on the following pages: *Hist. pisc.*, pp. 105–107, 118, 201, 125, 227–228, 236, 248, 249, 250, 252–254, 259, 260–262, 265, 266.

149 Baldner, *op. cit.* (note 137), f. 119r.

150 *Hist. pisc.*, pp. 201 and 125, cf. Baldner, *op. cit.* (note 137), f. 121r and f. 134r.

151 Baldner, *op. cit.* (note 137), f. 125r.

152 *Ibid.*, ff. 121v–122r. In his German history of fish, Gessner states that carp are sometimes born from chaos and dirt, and sometimes from seed and roe: see Conrad Gessner, *Fischbuch* (Christopher Froschauer, Zurich, 1563), pp. 164–165.

153 Original German: ‘und ich alles selbst in meiner Hand gehabt, dieselbige nach dem leben abmahlen laszen, und wird ein jdes bey Seinem Nahmen genännet, und so viel ich bey einem jeden gelernt, in Seiner Natur, Kurtzlich ausz eigener erfahrung daszelbe beschrieben’. Baldner, *op. cit.* (note 137), f. 3v.

154 Original German: ‘Und so mir Einer disze meine einfältige und geringe Arbeit, besser Verstehet, der wolle mirh, wo etwas gefehlt zu guth halten, Dann es von einem geringen Fischer und Weydman herkommet.’ *Ibid.*, ff. 3v–4r.

155 Original German: ‘So hab ich im Nahmen desz Herrn mein Netz und Fischerkarn ausz geworffen, und ein wenig von dem was ich erlernt, und in Dreysig Jahren dabey Studiert hab, ein wenig wollen anzeigen.’ *Ibid.*, f. 4r.

156 Original Latin: ‘Quo cibo utantur Salmones cum Autores diffentire videam, experientia consulenda est.’ *Hist. pisc.*, p. 192.

157 Birch, *op. cit.* (note 5), vol. 3, p. 425. See also Felicity Henderson, ‘Translation in the circle of Robert Hooke’, in *Translating early modern science* (ed. Sietske Fransen, Niall Hodson and Karl A. E. Enenkel), pp. 17–40 (Brill, Leiden, 2017), at p. 17.

158 Ralph Johnson, letter to John Ray, 16 April 1677, in Lankester, *op. cit.* (note 22), p. 128.

159 *Ibid*.

160 This historiography has become too vast to list exhaustively, but see, for example, Jim Bennett and Rebekah Higgitt (eds), *London 1600–1800: communities of natural knowledge and artificial practice*, special issue of *Brit. J. Hist. Sci.* **52**, 183–343 (2019); Harkness, *op. cit.* (note 34); Long, *op. cit.* (note 65); and Smith, *op. cit.* (note 65).

161 On the port of London as a (continued) source for faraway species, see Arthur MacGregor, ‘Patrons and collectors: contributors of zoological subjects to the works of George Edwards (1694–1773)’, *J. Hist. Coll.* **25**, 35–44 (2013), at p. 36.

162 Kathleen H. Ochs, ‘The Royal Society of London's history of trades programme: an early episode in applied science’, *Notes Rec. R. Soc. Lond.* **39**, 129–158 (1985), at p. 130.

163 Jasmine Kilburn-Toppin, ‘“A place of great trust to be supplied by men of skill and integrity”: assayers and knowledge cultures in late sixteenth- and seventeenth-century London’, in Bennett and Higgitt, *op. cit.* (note 160), pp. 197–223, at p. 222.

164 *Hist. pisc.*, p. 246; Leonard Mascall, *A Booke of Fishing with Hooke & Line, and of All Other Instruments There-unto Belonging* (John Wolfe, London, 1590), p. 8.

165 See, for example *Hist. pisc.*, pp. 219, 320.

166 D. Graham Burnett, *Trying Leviathan: the nineteenth-century court case that put the whale on trial and challenged the order of nature* (Princeton University Press, Princeton, 2007), pp. 95–145; Peter S. Alagona, ‘Species complex: classification and conservation in American environmental history’, *Isis* **107**, 738–761 (2016), at p. 753.

